# Diffuse panbronchiolitis: not just an Asian disease: Australian case series and review of the literature

**DOI:** 10.2349/biij.5.4.e19

**Published:** 2009-10-01

**Authors:** MP Anthony, S Singham, B Soans, G Tyler

**Affiliations:** 1 Department of Medical Imaging, John Hunter Hospital, Newcastle, Australia; 1 Department of Respiratory Medicine, John Hunter Hospital, Newcastle,Australia

**Keywords:** Diffuse panbronchiolitis, high resolution computed tomography, small airways disease, erythromycin; macrolide

## Abstract

Diffuse panbronchiolitis is a disease of obscure aetiology that is traditionally associated with Asian ethnicity. We propose that this disease also occurs in Caucasians and the incidence in this population is greater than currently recognised. We further propose that high resolution computed tomography (HRCT) and response to macrolide therapy should be relied upon to make this diagnosis without verification by lung biopsy. In most circumstances, obtaining a biopsy for histopathology is not practical, and the disease may then be mistaken for other more common airway diseases. Accuracy of diagnosis is important as untreated disease is associated with a poor prognosis, and effective treatment is available. We report four out of a series of cases as evidence that DPB is in fact more common in the Western population than is currently understood.

## INTRODUCTION

First described in 1969, diffuse panbronchiolitis (DPB) is a chronic inflammatory disease of the distal airways [1,2]. Patients present with a history of cough, large volume sputum production similar to bronchiectasis, exertional dyspnoea, and chronic sinusitis. Physical signs include rales and rhonchi. Lung function tests demonstrate a mixed obstructive/restrictive pattern. Chest radiograph features may be non-specific, with the presence of ill-defined small nodular shadows, hyperinflation, and in later stages, bronchiectasis. HRCT features classically include small centrilobular nodules and branching linear opacities – the ‘tree-in-bud’ (TIB) appearance. Treatment consists of macrolide antibiotic in low dosage for 3 to 6 months.

## CASE REPORTS

### Case 1

A 64-year-old Caucasian female non-smoker presented in June 2004 with a 20-year history of non-productive cough. There was no significant past medical or exposure history. Previous therapeutic trials of a combination inhaler containing budesonide and efermoterol, and omeprazole had been ineffective.

Clinical examination, spirometry and flow volume loop were normal. Forced expiratory volume in 1 second (FEV1) was 96% predicted, and forced vital capacity (FVC) was 125% predicted. A bronchial provocation with hypertonic saline challenge revealed a PD20 greater than 20 milli-litres (normal). Serum immunoglobulin levels were normal.

HRCT demonstrated mild bronchiectasis in the lower lobes, and diffuse centrilobular nodules. There was a scattered TIB appearance.

There was complete resolution of symptoms when treated with erythromycin 250mg twice daily for three months.

**Figure 1:**
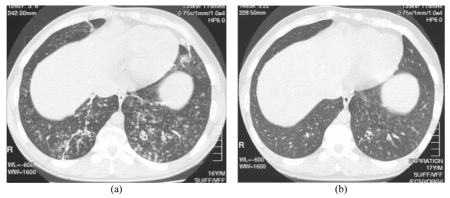
HRCT images of the lower chest in a 16 year-old boy (Case 2) at presentation (A), and 8 weeks later after a 6-week course of erythromycin 250mg twice daily. The bilateral bronchiectasis and prominent centri-lobular nodules with a tree-in-bud pattern shows marked improvement on the follow-up scan

### Case 2

A 16-year-old Caucasian Australian-born boy presented in March 2004 with a chronic cough productive of yellow sputum. The cough began in early childhood and was initially intermittent but became more severe and persistent around the age of 11 years. There was no associated wheeze or breathlessness. The severity of the cough gradually increased. His symptoms were attributed to asthma and he received asthma therapy including intermittent oral steroids without benefit for several years. There was no history of smoking, dust nor fume exposure.

He had a barrel chest, and had widespread bilateral crackles. There were no signs of pulmonary hypertension, and no clubbing.

Spirometry showed mixed restrictive and obstructive change with an FEV1/FVC of 2.4/3.34 litres without acute reversibility (predicted 3.93/4.26). Sputum cultures grew *haemophilus influenzae*. No acid fast bacili was grown from repeated sputum cultures. There was a mild elevation in white cell count, but other blood tests were normal.

HRCT demonstrated bilateral bronchiectasis, and diffuse centrilobular nodules predominantly affecting the mid and lower zones. There was diffuse TIB appearance.

No improvement in symptoms resulted when treated with augmentin. A 6-week trial of erythromycin 250mg twice daily led to resolution of symptoms. There was an approximately 80% clearance of the above-mentioned HRCT findings on repeat examination. Spirometry improved to an FEV1/FVC of 4.36/4.94 litres.

### Case 3

A 65-year-old Caucasian Australian-born man presented in October 2004 with a 6-year history of cough productive of mucoid sputum, wheeze, breathlessness and worsening pan-sinusitis. There was a history of hay fever and gastro-oesophageal reflux. He had experienced short-term improvement with a number of courses of antibiotics relapsing after each was complete. There was no history of smoking, occupational dust or fume exposure. Presenting symptoms had been attributed to asthma but had not responded to asthma therapy.

Clinical examination revealed generalised wheezing, but no other abnormality was detected.

Spirometry showed mild airflow obstruction with improvement post bronchodilator. FEV1/FVC pre bronchodilator 1.96/3.0 litres, and 2.28/3.82 litres post bronchodilator (predicted 2.85/3.64 litres).

HRCT demonstrated mild basal bronchiectasis and basal TIB.

There was an improvement in spirometry (FEV1/FVC = 2.68/3.75 without bronchodilator response) and complete resolution of his symptoms after a 3-month course of erythromycin 250mg twice daily.

### Case 4

A 73-year-old Caucasian Australian-born woman presented in August 2003 with 6 months of cough productive of mucopurulent sputum, but no systemic symptoms. There was no significant past medical history, and no history of environmental exposures or of smoking. The cough had not responded to several courses of oral antibiotics including ciprofloxacin.

Clinical examination and spirometry was normal (FEV1 129 % predicted, FVC 130% predicted). Sputum cultures grew *Pseudomonas aeruginosa* and *Mycobacterium avium* complex (MAC) in 1 out of 3 samples. Bronchoscopic samples grew *Pseudomonas aeruginosa* only. There was no immunoglobulin deficiency.

The chest x-ray demonstrated changes of right middle and lower lobe bronchiectasis and large lung volumes. HRCT showed bronchiectasis in the middle and lower lobes, and some atelectasis. There were diffuse centrilobular nodules. The left lower lobe also demonstrated TIB appearance.

A 2-week course of ciprofloxacin and prednisone did not improve her symptoms. A 3-month course of erythromycin 250mg twice daily led to resolution of her cough.

## DISCUSSION

DPB is a chronic inflammatory disease of obscure aetiology affecting the distal airways, predominantly at the transition zone between the respiratory bronchioles and alveoli [1,2].

The disease typically occurs in the 2^nd^ to 5^th^ decade of life (average onset 40 years). The male: female ratio is 1.4:2.1. Two-thirds of patients are non-smokers and patients have no particular history of inhalation of toxic fumes [3]. Whilst initially almost exclusively reported in Japan [4] there have now been sporadic cases reported in every continent [5]. The pathogenesis is still unclear, and thought to be related to both genetic and environmental factors. Sugiyama *et al.* [3] showed that 63% of patients possessed the HLA-Bw54 antigen. She *et al.* demonstrated increased frequency of class I HLA-A11. HLA-associated major histocompatibility genes are thought to be located in the HLA class I region between the HLA-A and HLA-B loci on chromosome 6p21.3 [6, 7].

DPB is often confused with other airway diseases characterised by chronic productive cough [8], including bronchiectasis, chronic bronchitis, and cystic fibrosis [9]. Due to this confusion and the importance of an accurate diagnosis, the diagnosis of DPB has often relied upon histopathologic confirmation. Pathologic criteria include 1) diffuse, bilateral, chronic inflammatory airway disease; 2) predominant involvement of the walls of respiratory bronchioles and adjacent centrilobular regions; and 3) interstitial accumulation of foamy macrophages [10]. Typical features seen include thickening of the walls of the respiratory bronchioles, transmural and peribronchial infiltration by lymphocytes, plasma cells, and histocytes [11]. The inflammatory infiltrate destroys the bronchiolar epithelium and extends to peribronchiolar spaces, but most of the alveoli are unaffected [12]. The characteristic ‘unit lesion’ of DPB described by Kitaichi [13] incorporates some of these features, and is considered to histologically distinguish DPB from a number of other entities including chronic bronchitis, cystic fibrosis, Mycoplasma pneumonia and cryptogenic organising pneumonia [14].

There are obvious restraints in the ability to always obtain a biopsy for diagnosis and an increasing reliance on clinical and radiologic findings should be encouraged. A plain chest X-Ray (CXR) reveals bilateral, diffuse, small (<5mm) nodular shadows predominantly in the lower field of the lung with hyperinflation. In advanced cases, ring-shaped or tram-line shadows appear, indicating bronchiectasis.

By contrast, the HRCT features of diffuse panbronchiolitis – given the appropriate clinical setting – can be near pathognomonic [15], and are more representative of the pathologic process [16]. In advanced stages, multiple cystic lesions predominate in the lower lung fields and are accompanied by dilated proximal bronchi showing the appearance of extensive bronchiectasis. These findings extend from the respiratory bronchioles to the proximal airways [17]. Nodular shadows are distributed in a centrilobular fashion, often extending to small, branching linear areas of attenuation (“TIB” pattern). Peripheral air trapping is usually confirmed in expiratory films. Dilatation of airways and bronchial wall thickening are present. Mosaic oligoemia is usually absent [12].

The natural history of DPB is progressive respiratory failure marked by chronic and/or frequent episodes of superimposed bacterial infection. The capacity for gas exchange is reduced, which causes progression of hypoxaemia, and later, hypercapnia. Pulmonary hypertension develops and is associated with the development of cor pulmonale and ultimately death within 5 years for up to 20% of patients [13]. However, the cases described above may lead to the conclusion that the disease is less severe in Caucasians . These patients had persistent symptoms for several years without serious impairment of lung function.

Appropriate management with a low-dose macrolide antibiotic such as erythromycin (200-600mg daily), is well recognised to significantly alter the course of the disease [18], resulting in improved symptoms and pulmonary function tests, reduced hypoxaemia, improved radiographic appearances, and usually cure and improved survival [19]. It has been found that both serum and sputum erythromycin levels with treatment are actually below the minimum inhibitory concentration for the common superinfecting organisms, and it is postulated that the mechanism of action of erythromycin is related to anti-inflammatory and immunomodulatory effects [20, 21], rather than to its antimicrobial properties [22].

Nishimaki *et al.* propose that anti-inflammatory effects, including the inhibition of GM-CSF production from monocytes, lung fibroblasts and bronchial epithelial cells and the acceleration of neutrophil apoptosis by the action of macrolide therapy, is what leads to improved survival [23]. Studies show an interference with neutrophil chemotaxis by erythromycin, and resultant lower neutrophil levels in broncho-alveolar lavage fluid samples. In addition, macrolides reduce the presence of activated T-lymphocytes at the site of inflammation, promoting T-cell apoptosis via the down-regulation of anti-apoptotic Bcl-2 protein family in DPB which are present in high levels in such patients [24].

## CONCLUSION

A lack of familiarity with the diagnosis of DPB in Western countries has led to under-diagnosis and failure to treat such cases [25, 26]. In view of the dramatic effectiveness of treatment, and that the treatment itself is very specific, the diagnostic features of DPB need to be recognised early. Fitzgerald *et al.* [5] suggest that if the appropriate clinical, physiologic, and radiological features are present, then lung biopsy is usually unnecessary, and a therapeutic trial of a macrolide antibiotic is appropriate. The reported cases illustrate that this approach is successful. These cases also highlight that, in Caucasians, the disease may be less severe. However, treatment can lead to symptom control. Improved awareness of the disease should lead to higher rates of diagnosis and, therefore, improved treatment of such patients.

## ABBREVIATIONS

DPB: diffuse panbronchiolitis
HRCT: high resolution computed tomography
TIB: tree in bud
FEV1: forced expiratory volume in 1 second
FVC: forced vital capacity
CXR: chest X-Ray
